# Association of prenatal and postnatal exposure to air pollution with clinically diagnosed attention deficit hyperactivity disorder: a systematic review

**DOI:** 10.3389/fpubh.2024.1396251

**Published:** 2024-05-24

**Authors:** Jinzhu Zhao, Tianyi He, Feng Wang, Wei Liu

**Affiliations:** ^1^Division of Child Healthcare, Department of Pediatrics, Tongji Hospital, Tongji Medical College, Huazhong University of Science and Technology, Wuhan, China; ^2^Tongji Medical College, Huazhong University of Science and Technology, Wuhan, Hubei Province, China; ^3^Department of Public Health, Tongji Hospital, Tongji Medical College, Huazhong University of Science and Technology, Wuhan, Hubei Province, China

**Keywords:** air pollution, ADHD, prenatal, postnatal, systematic review

## Abstract

Attention deficit hyperactivity disorder (ADHD), a prevalent neurodevelopmental disorder in children, originates from a multifaceted interplay of genetic, neurological, and environmental factors. Recent studies have increasingly concentrated on environmental determinants, notably air pollution, and their impact on the risk of developing ADHD. Additionally, previous research has often conflated clinically diagnosed ADHD cases with instances of mere ADHD-like symptoms, a methodology that can introduce bias and obscure the true relationship between environmental factors and ADHD. To address this oversight, our systematic review meticulously investigates the relationship between both prenatal and postnatal exposures to particular air pollutants and strictly clinically diagnosed ADHD. Our comprehensive review encompassed 801 studies from PubMed, Cochrane Library, Web of Science, and Embase databases, out of which eight met our rigorous inclusion criteria. The Newcastle-Ottawa Scale (NOS) was utilized to gauge quality and bias. Our review found substantiated the connection between prenatal exposure to PM_2.5_ and NO_x_ and a heightened risk of ADHD, while exposure to PM_10_ during the prenatal stage was not associated with ADHD. These findings hint at varied health impacts from different particulate matters and the prospect of gender-specific susceptibilities to such exposures. We also identified an association between postnatal exposure to PM_2.5_, PM_10_, and NO_2_ and an increased ADHD risk, underlining the potential neurodevelopmental harms from early exposure to these pollutants. These relationships, seemingly intricate and potentially dose-dependent, underscore the need for more detailed scrutiny. The unique value of our review is in its detailed exploration of the association between specific air pollution exposures and clinically diagnosed ADHD. Our findings offer much-needed clarity in this complex domain and emphasize the importance of future research to standardize exposure and outcome metrics, probe potential mechanisms, and reduce bias and heterogeneity.

## 1 Introduction

Attention Deficit Hyperactivity Disorder (ADHD) is a complex neurodevelopmental disorder that usually begins in childhood and often persists into adulthood. Characterized by inattention, hyperactivity, and impulsivity, ADHD symptoms significantly differ from expected developmental milestones and affect approximately 2 to 7% of children and adolescents worldwide (1). Males are diagnosed more frequently than females, with clinical studies showing a male-to-female ratio of 4:1, and population studies indication a ratio of 2.4:1 (2). The disorder impacts social, educational, and occupational settings, negatively affecting individuals’ daily life and overall well-being (3).

ADHD is known for its behavioral variability, multiple etiological factors, and diverse developmental trajectories, often co-occurring with other conditions, and showing varied treatment responses (4). Genetic and environmental factors are recognized as crucial in ADHD’s etiology, with air pollution emerging as a significant environmental risk factor (5). Air pollution has emerged as a significant environmental factor associated with the development of ADHD. Building on existing research that has identified maternal health and prenatal conditions as contributors to ADHD, attention is increasingly turning toward environmental pollutants (6). There is compelling evidence linking air pollution exposure, particularly prenatal, to heightened ADHD risk, with pollutants crossing from maternal blood to the fetus (7). Pollutants such as particulate matter (PM_2.5_), nitrogen oxides (NO_x_), and polycyclic aromatic hydrocarbons (PAHs) are implicated in oxidative stress and inflammation, key processes affecting neurological health (8). They can prompt the production of reactive oxygen species, activate brain microglia, and provoke inflammatory responses that compromise blood–brain barrier integrity and disrupt neural cell development (9, 10). Air pollution is also associated with apoptosis in neural cells and disruptions in the differentiation of neural stem cells, both vital for brain development and function (11). This research supports the hypothesis that exposure to pollutants during critical developmental periods contributes to the rise of developmental disorders worldwide (12). Consequently, there is a pressing need for further investigation into the environmental risk factors for ADHD to inform strategies for reducing its incidence and severity.

Research demonstrates that prenatal and early life exposure to pollutants can adversely affect brain volume and cognitive functions, leading to increased behavioral problems. Specifically, exposure to PM_2.5_ during pregnancy is associated with reduced white matter in the brain’s left hemisphere, which may increase the risk of ADHD symptoms (13, 14). Despite the profound implications of air pollutant exposure on neurodevelopment, potentially resulting in severe lifelong disabilities, its recognition remains limited, often referred to as a “silent killer” (4). Challenges in quantifying these effects and mixed evidence regarding their association have contributed to this lack of recognition, with only a few studies finding no link between prenatal and postnatal air pollutant exposure and ADHD (15). The inconsistency in findings underscore the need for a rigorous investigation into the effects of prenatal exposure on ADHD development, necessitating more comprehensive data to better understand these relationships.

Additionally, recognizing the distinction between clinical diagnoses of ADHD and observations of ADHD-like behaviors is crucial in epidemiological research. Often, systematic reviews and meta-analyses conflate clinically diagnosed individuals with those exhibiting similar symptoms. This overlap introduces heterogeneity and bias, which can obscure the relationship between prenatal exposure to air pollution and the clinical diagnosis of ADHD. For more accurate assessments, it is essential to focus on subjects who have undergone rigorous, clinically validated diagnostic procedures for ADHD. These comprehensive evaluations incorporate behavioral ratings, clinical interviews, neurocognitive assessments, and information from multiple informants, such as parents, teachers, and clinicians. Moreover, these evaluations necessitate the exclusion of other potential etiological factors or comorbid conditions that could present with ADHD-like symptoms. To address the heterogeneity introduced by differences in diagnostic procedures, it is crucial to include the standardize diagnostic criteria and processes within the study, which helps ensure that variations in results are attributable to the studied factors. Exclusive reliance on singular assessment tools, such as the Behavior Checklist (CBCL) (16, 17), Continuous Performance Test (CPT) (18), Wide Range Assessment of Memory and Learning (WRAML) (19), and the strengths and difficulties questionnaire (SDQ) (15, 20, 21), is insufficient for a definitive diagnosis of ADHD. Each tool, while valuable, forms only a part of the comprehensive assessment required for a robust clinical diagnosis. Furthermore, research often does not differentiate between prenatal and postnatal exposure periods, overlooking the differential impacts of air pollution across developmental stages. A nuanced approach is necessary to distinguish between prenatal and postnatal influences, clarifying their distinct contributions to the disorder’s etiology (15).

Our meta-analysis aims to refine the understanding of the link between prenatal and postnatal air pollution exposure and clinically diagnosed ADHD amid significant heterogeneity in existing research. We conducted a systematic review and synthesis of studies that meet stringent inclusion criteria, specifically those offering definitive clinical diagnoses of ADHD. By elucidating the association between air pollution and ADHD, we aim to inform future research directions for more targeted investigations.

## 2 Methods

This systematic review adheres strictly to the guidelines set forth by the Preferred Reporting Items for Systematic Reviews and Meta-Analyses (PRISMA) statement, ensuring a rigorous and comprehensive approach. For detailed adherence and verification, the complete PRISMA checklist can be found in Appendix 1.

### 2.1 Literature search

**Figure 1:**
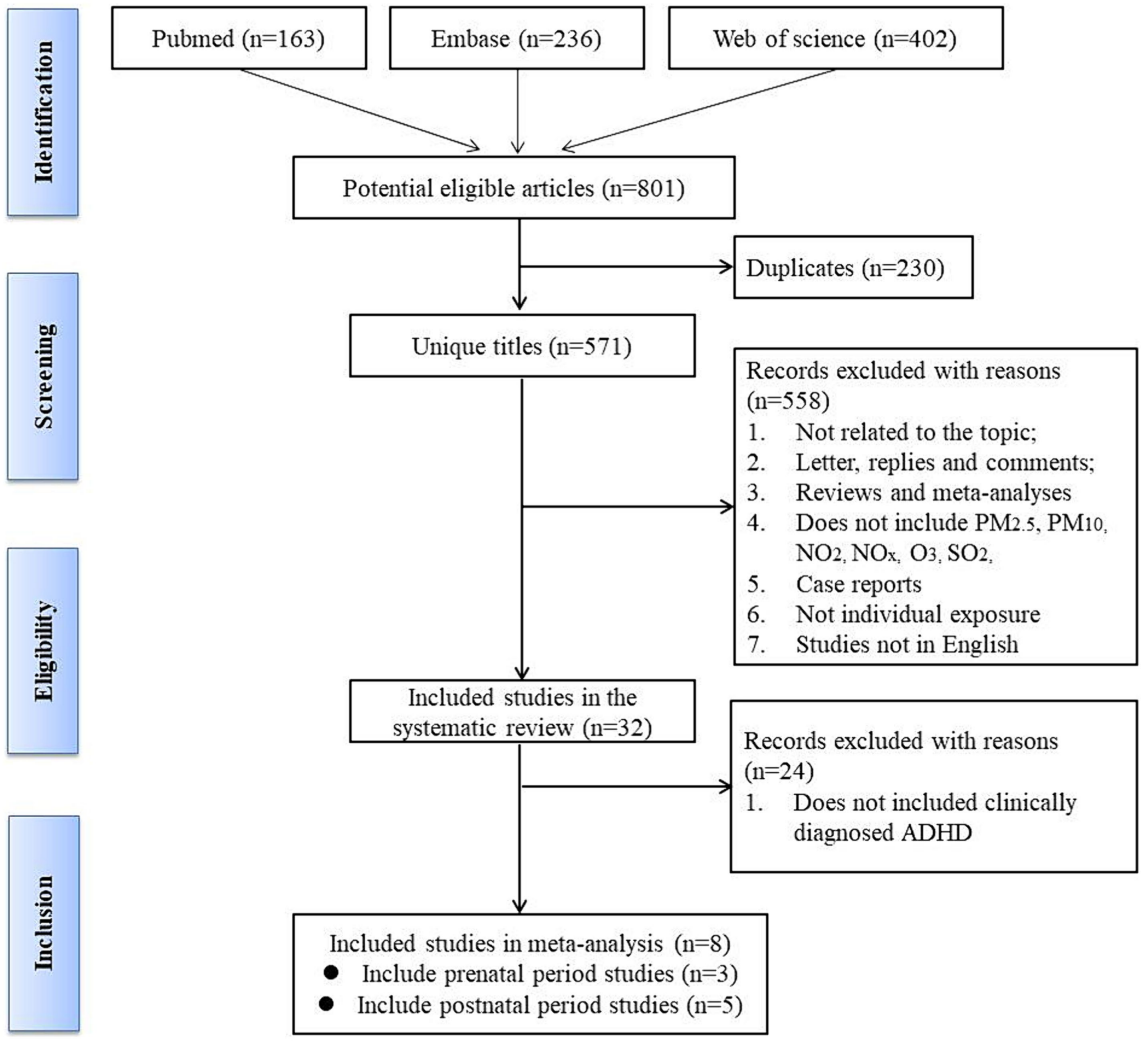
Flow diagram of the selection process in the systematic review.

The protocol of this study was registered in PROSPERO (No. CRD42023417136). We conducted a comprehensive review of studies that explored the association between air pollutants and ADHD. To achieve this, we searched the PubMed, Cochrane Library, Web of Science, and Embase databases for original articles. Our search strategy employed a combination of Medical Subject Headings (MeSH) pertaining to ADHD and maternal exposure to air pollution during all three trimesters including the prenatal and postnatal period. We included five primary air pollutants: carbon monoxide (CO), NO_x_, ozone (O_3_), PM, and sulfur oxides (SO_2_). And we restricted our search to studies with abstracts published in English and having publication dates between January 1, 2007, and April 30, 2023. To identify studies not discovered through our database search strategy, we manually searched for additional articles by examining reference lists from all publications meeting the inclusion criteria and relevant systematic reviews. Our search strategy is depicted in Figure 1 and provided in Appendix 2.

### 2.2 Study eligibility criteria

We determined the eligibility of articles for this study using the following inclusion criteria: (1) observational study design, encompassing case–control and cohort studies; (2) study participants including children and/or their mothers, and the children aged 18 years or younger; (3) clinically diagnosed ADHD; (4) prenatal and postnatal exposure to outdoor air pollutants, including NO_x_, NO_2_, SO_2_, PM_10_, PM_2.5_, and O_3_, measured during conception and pregnancy; and (5) availability of information on sample size and air pollution. We excluded studies based on the following criteria: (1) those employing animal models; (2) no clinical diagnosis of ADHD, such as hyperactivity or attention problems, behavioral issues, etc.; (3) full articles not published in English; and (4) abstracts, case reports, comments, reviews, conference proceedings, or book chapters.

### 2.3 Quality of assessment

We utilized the Newcastle-Ottawa Quality Assessment Scale (NOS) to evaluate the quality of the articles and assess the risk of bias. The NOS model comprises eight items focusing on selection bias (4 items), comparability bias (1 item), and outcome bias (3 items; refer to Supplementary Figure 1). Studies were allocated a maximum of 1 point for each of the seven items, and 2 points specifically for comparability, resulting in a maximum score of 9 points. Higher scores signified a lower risk of bias. We established a cut-off score of 7 using the NOS.

### 2.4 Data extraction

The comprehensive literature search and analysis were carried out by two independent authors, JZ and TH. Their thorough examination of the extensive body of literature on the subject adhered to predefined selection criteria, aiming to ascertain the relevance and appropriateness of each article for inclusion in the study. We employed a rigorous, transparent, and collaborative deliberation process to enhance the methodological robustness of our study. All inconsistencies among reviewers were meticulously discussed during structured team meetings aimed at achieving consensus. If a consensus proved elusive, we involved a third contributing author, WL, to mediate and resolve the impasse. This approach not only ensured the incorporation of diverse perspectives but also fostered a balanced and comprehensive evaluation of the data. Such collaboration significantly strengthened the credibility and reliability of our findings.

Upon reaching a unanimous agreement, the JZ and FW proceeded with a rigorous review of the selected articles. Key data points were carefully extracted from each study, including the first author’s name, the year of publication, the year of follow-up, the country of origin, the demographic characteristics of the study population, the sample size, the exposure variable, the methodologies applied for ADHD assessment, the types and sources of pollutants examined, the key findings, and any observed variations or anomalies.

## 3 Results

### 3.1 Search results and study characteristics

**Figure 2:**
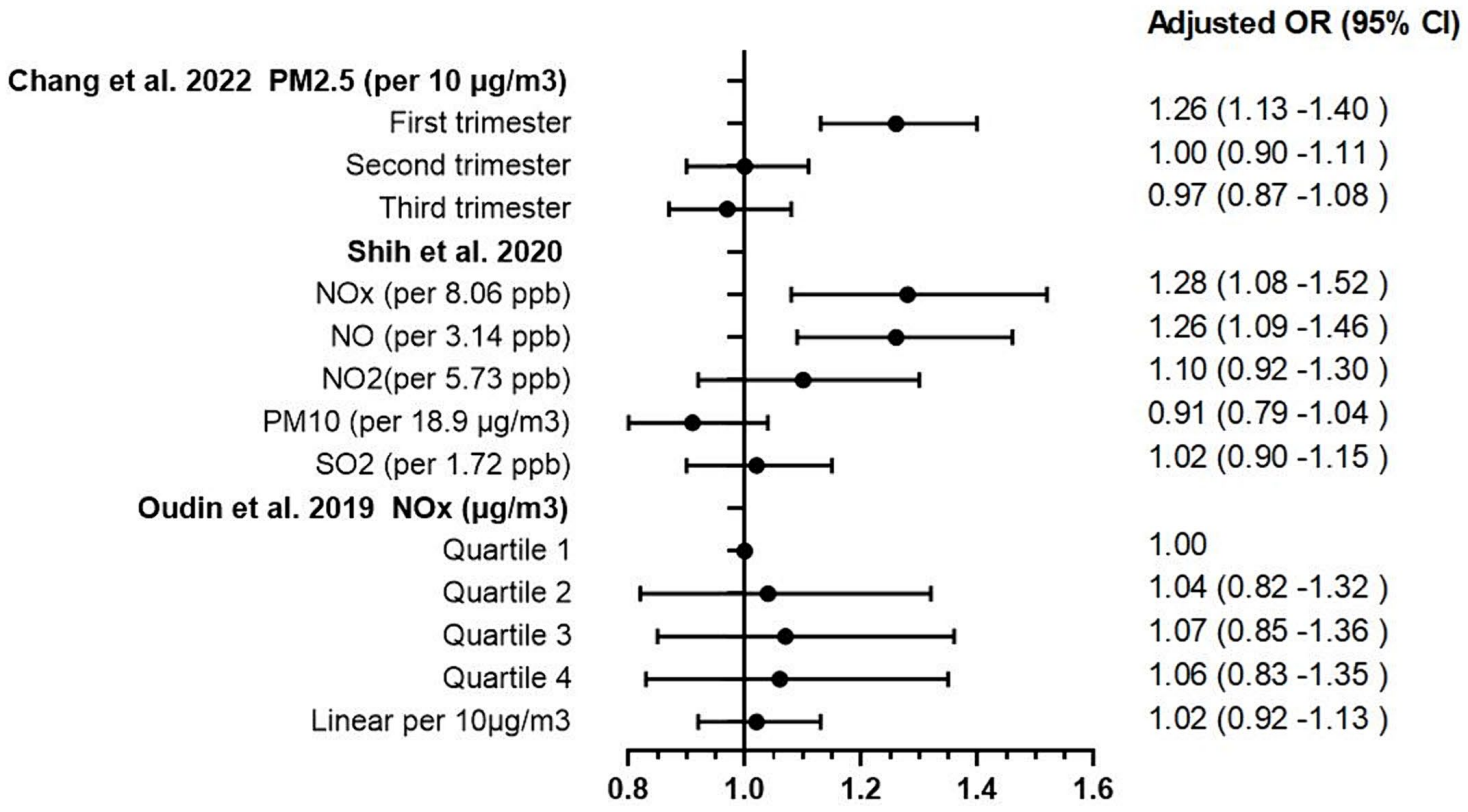
Odd ratios forest plot of association between prenatal exposure to pollutant and ADHD.

**Figure 3:**
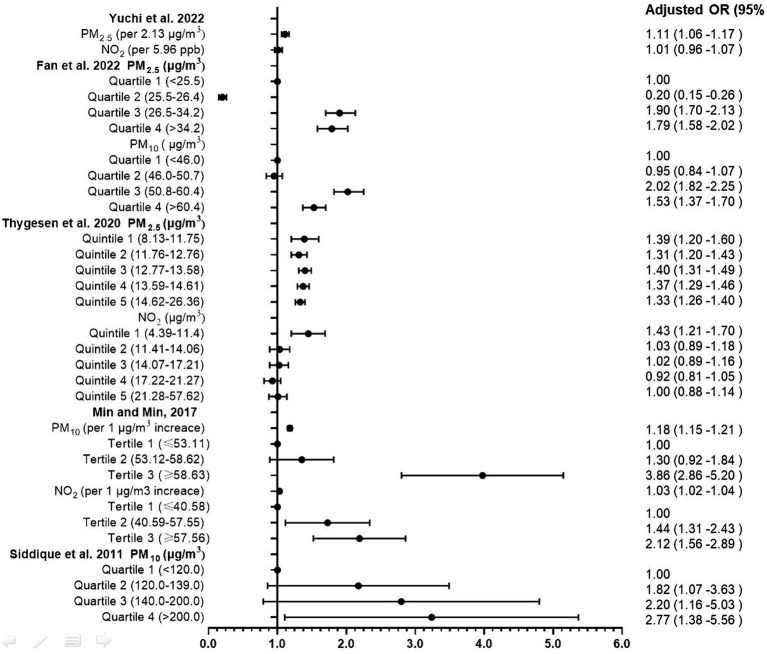
Odd ratios forest plot of association between postnatal exposure to pollutant and ADHD.

The process of article selection for inclusion in this review is depicted in Figure 1. A total of 801 articles were initially identified from database searches. After duplicate removal, 571 records remained. The initial screening of titles and abstracts further reduced the pool to 32 studies. Full-text evaluations of these 32 articles resulted in a final count of 8 published studies that satisfied our inclusion criteria. Methodological heterogeneity across studies, including disparate time windows of exposure, divergent approaches to exposure assessment, and variability in outcome measurement, precluded the feasibility of a meta-analysis. The main characteristics of the studies included in this review are summarized in Tables 1, 2 and Supplementary Table S1 (see Appendix 3), in order by date of publication. Figure 2 shows the results of the eight articles with reported odds ratios. The forest plots depict the odd ratios associated with prenatal exposure to NO_x_, NO, NO_2_, SO_2_, PM_2.5_, and ADHD in children, as provided by multiple studies. Similarly, the studies offering odd ratios related to postnatal exposure to PM_2.5_, PM_10_, NO_2_, and ADHD in children are also represented in forest plots (Figure 3). Essentially, the forest plot graphs offer a vivid portrayal of the varied associations.

**Table 1:** Data extracted from articles on prenatal air pollutant exposure and ADHD.

Author & year	Country	Study design	Exposure period	Study pollution	Age group	n ADHD/ n total	Exposure (Air pollutants)	How exposure measured	Diagnostic criteria	Adjustment of risk	Main findings
Chang et al. 2022 (22)	Taiwan	Cohort study	Prenatal and postnatal period	Children from the Taiwan Maternal and Child Health Database (TMCHD)	1–5 years	9294/440258	PM_2.5_	Satellite-based estimation model	ICD-9	Sex maternal age at delivery socioeconomic status maternal smoke maternal drug abuse childhood iron deficiency anemia	An association between PM_2.5_ exposure during pregnancy and offspring ADHD
Shih et al. 2020 (23)	Taiwan	Cohort study	Pregnancy	Children from the Taiwan Birth Cohort Study (TBCS)	0–8 years	374/16376	PM_10_ NOx SO_2_ NO NO_2_	Spatial interpolation	DSM-IV-TR	Sex urban residence birth in summer low annual household income average ambient temperature	An association between NO exposure during pregnancy and offspring hyperactivity
Oudin et al. 2019 (24)	Southern Sweden	Cohort study	Pregnancy	Children from Maternal Air Pollution in Southern Sweden (MAPSS) database	5–17 years	718/48571	NO_x_	Gaussian dispersion model	DSM ICD-10	Gender of child maternal age parity maternal smoking maternal BMI maternal education disposable income maternal country of birth	No association between NO_x_ exposures and ADHD

ICD-9, International Classification of Diseases, 9th Edition; ICD-10, International Classification of Diseases, 10th Edition; DSM-IV-TR, Diagnostic and Statistical Manual of Mental Disorders, 4th Edition, Text Revision; DSM, Diagnostic and Statistical Manual of mental disorders.

**Table 2:** Data extracted from articles on postnatal air pollutant exposure and ADHD.

Author & year	Country	Study desigh	Exposure period	Study pollution	Age group	n ADHD/n total	Exposure (Air pollutants)	How exposure measured	Diagnostic criteria	Adjustment of risk	Main Findings
Yuchi et al. 2022 (25)	Canada (Metro Vancouver)	Cohort study	Postnatal period	Children from the administrative datasets (Registration, Physician Visit, and Hospital Discharge data) from the British Columbia Ministry of Health Services	3–10 years	1217/28797	PM_2.5_ NO_2_	Land-use regression models	ICD-9 ICD-10	Maternal age paternal age maternal BMI maternal parity birth weight season of birth gestational age infant sex education household income residential instability material deprivation dependency and ethnic concentration	A significant association was observed between PM_2.5_ and ADHD; no association was identified between NO_2_ and ADHD
Fan et al. 2022 (26)	Taiwan	Cohort study	Postnatal period	Children from the Taiwan National Health Insurance Research Database (NHIRD)	0–18 years	2856/98177	PM_2.5_ PM_10_	Temporal and spatial resolution	ICD-9-CM ICD-10-CM	Sex Age urbanization level comorbidities	Exposure to particulate matter, specifically PM_2.5_ and PM_10_, was linked with a significantly increased risk of ADHD.
Thygesen et al. 2020 (27)	Danish	Cohort study	Postnatal period	Children from the Danish Civil Registration System (CRS)	5–16 years	19,045/809654	PM_2.5_ NO_2_	THOR modeling system	ICD-10-DCR	Age calendar year sex mother’s and father’s level of education and income	Exposure to NO_2_ and PM_2.5_ was associated with a significantly increased risk of ADHD
Min and Min, 2017 (28)	Korea	Cohort	Postnatal period	Children from the National Health Insurance Service-National Sample Cohort (NHIS-NSC)	10 years	314/8936	NO_2_ PM_10_	Ambient air monitoring station and GIS tools	DSM-IV-TR	Gender metropolitan area household income medical history	A significant association was observed between ADHD and exposure to PM_10_ and NO_2_
Siddique et al. 2011 (29)	India (Delhi and West Bengal)	Cross sectional	Postnatal period	Children from different areas of National Capital Region of Delhi and rural areas of Uttaranchal and West Bengal	9–17 years	Urban:107/969 Rural:23/850	PM_10_	Fixed-site monitoring stations were established at 60 urban and 60 rural households.	DSM-IV	Age Gender SES Prenatal smoking	PM_10_ exposure was found to be positively associated with the ADHD.

ICD-9, International Classification of Diseases, 9th Edition; ICD-10, International Classification of Diseases, 10th Edition; DSM-IV-TR, Diagnostic and Statistical Manual of Mental Disorders, 4th Edition, Text Revision; DSM, Diagnostic and Statistical Manual of mental disorders; ICD-9-CM, International Classification of Diseases, Ninth Revision, Clinical Modification; ICD-10-CM, International Classification of Diseases, Tenth Revision, Clinical Modification; ICD-10-DCR, The ICD-10 Diagnostic Criteria for Research.

### 3.2 Prenatal exposure to air pollution and ADHD studies

Table 1 offers a detailed exploration of prenatal exposure to air pollution and its potential implications on ADHD. Emphasizing this connection, three significant studies that used clinical ADHD diagnostic criteria, such as ICD-9/ICD-10 or DSM-IV, have been spotlighted. They collectively included a robust sample size of 505,205 participants, out of which 10,386 were diagnosed with ADHD. With data gathered from two diverse regions, Taiwan and Southern Sweden.

Chang et al. (22) undertook an extensive exploration of the implications of PM_2.5_ exposure during both prenatal and postnatal periods on ADHD occurrence. This Taiwanese birth cohort study traced a significant sample of 425,736 infants from conception to their first 5 years of life. Results indicated a striking rise in ADHD risk following PM_2.5_ exposure in prenatal period with an Adjusted hazard ratio (HR) of 1.26 per 10 μg/m^3^ increase in PM_2.5_ exposure during the first trimester (95% CI 1.13–1.40). The strength of these findings is further bolstered by the comprehensive controls for potential confounding factors and the use of a satellite-based PM_2.5_ prediction model to accurately estimate PM_2.5_ concentrations.

Meanwhile, Shih et al. (23) turned their attention to the effects of prenatal exposure to traffic-related air pollution, drawing data from 16,376 full-term singleton mothers and their children up until the age of eight in Taiwan. Their findings offer significant insights, revealing that prenatal exposure to traffic-related air pollutants, notably NO_x_ (adjusted OR 1.28 per 8.06 ppb increase, 95% CI 1.08–1.52), NO (adjusted OR 1.26 per 3.14 ppb increase, 95% CI 1.08–1.52) were linked to an increased risk of ADHD in children. Importantly, they observed a stronger correlation in boys than girls, signaling a potential gender disparity in air pollution’s impact on ADHD development.

Oudin et al. (24) conducted an extensive study of 129,127 children born between 1998 and 2007 in Skåne County, Sweden, yielded different results. They found that elevated levels of prenatal NO_x_ exposure (as measured in quartiles, with adjusted ORs ranging from 1.04 to 1.06, and 95% CIs falling between 0.82 and 1.35) were associated with an increased risk of autism spectrum disorder (ASD), but not ADHD. This inconsistency underscores the intricate and multifaceted nature of the association between air pollutants and neurodevelopmental disorders, illuminating the need for further investigations in this domain.

The Supplementary Table S1 comprises 26 studies that examined the prenatal and postnatal exposure to air pollution and its link to ADHD-like behavior.

### 3.3 Postnatal exposure to air pollution and ADHD studies

Table 2 meticulously illustrates the complex relationship between postnatal exposure to air pollutants and ADHD. This association is substantiated by five detailed studies that utilized internationally recognized diagnostic criteria for ADHD, such as ICD-9/ICD-10 and DSM-IV. Collectively, these studies encapsulated a substantial cohort of 947,383 participants, from which 23,562 individuals were diagnosed with ADHD. These studies amalgamate and analyze data garnered from diverse geographical locales, including Metro Vancouver in Canada, Taiwan, Denmark, Korea, Barcelona in Spain, and Delhi and West Bengal in India.

Yuchi et al. (25) embarked on a comprehensive exploration, aiming to investigate the connection between prenatal exposure to PM_2.5_, NO_2_ with the risk of ADHD in offspring. Leveraging a 10-year dataset (2000–2010) from the Medical Services Plan involving children and their mothers, their robust study led to crucial revelations. Upon examining 1,217 newly diagnosed ADHD cases, a significant correlation emerged between PM_2.5_ exposure during the child’s initial 3 years and the subsequent risk of ADHD (HR: 1.11 for every 2.13 μg/m^3^ increase), bolstering the evidence for the neurodevelopmental implications of early life exposure to air pollutants.

Fan et al. (26) drew upon Taiwan’s National Health Insurance Research Database and environmental data to elucidate the implications of early childhood exposure to PM_2.5_ and PM_10_ on ADHD risk. Their investigation spanned a cohort of 98,177 participants tracked over an average duration of 14.7 years. Their findings indicated a perceptible increase in ADHD risk among children exposed to these pollutants, with a distinctive dose–response relationship demonstrated through quartile analysis.

Thygesen et al. (30) conducted a large-scale, population-based cohort study, meticulously analyzing children born in Denmark from 1997 to 2013 over a period of up to 16 years. They reported a significant correlation between exposure to NO_2_ and PM_2.5_, particularly from traffic-related pollution during the first year of life, and an increased ADHD risk. Their quintile analysis demonstrated a dose–response pattern.

In a decade-long longitudinal study, Min and Min (28) delved into the association between PM_10_ and NO_2_ exposure and ADHD in childhood among 8,936 participants. The study revealed a noteworthy correlation between infancy exposure to these pollutants (Adjusted OR: 1.18 for PM_10_ and 1.03 for NO_2_ per 1 μg/m3 increase) and an elevated risk of developing ADHD, underscoring the potential for long-term impacts of early environmental exposures on child health.

Lastly, Siddique et al. (29) investigated the correlation between vehicular pollution, specifically PM_10_, and ADHD prevalence among children in Delhi, India. Their analysis identified PM_10_ exposure as a significant risk factor for ADHD, as evidenced by the increased odds ratios across quartiles of exposure levels.

### 3.4 Quality assessment

In assessing the quality of the selected studies in our review, we applied the NOS. The Each component carries a certain number of ‘stars’ or points, with a maximum attainable score of nine stars. In our analysis, all the chosen cohort and cross-sectional studies garnered a minimum of 7-star rating on the NOS (Tables 3, 4), indicating a ‘good’ quality categorization as per common consensus. This rating is indicative of the high methodological rigor employed in these studies, including careful selection of exposed and non-exposed cohorts, adequate consideration of confounding factors, and robust methods for outcome or exposure ascertainment.

**Table 3:** NOS quality assessment for prenatal exposure to ADHD.

Paper	Selection (Max 4 stars)	Comparability (Max 2 stars)	Outcome (Max 3 stars)	Total score
Chang et al., 2022	★★★★	★★	★★★	9/9
Shih et al., 2020	★★★★	★★	★★★	9/9
Oudin et al., 2019	★★★★	★★	★★★	9/9

**Table 4:** NOS quality assessment for postnatal exposure to ADHD.

Paper	Selection (Max 4 stars)	Comparability (Max 2 stars)	Outcome (Max 3 stars)	Total score
Yuchi et al., 2022	★★★★	★★	★★	8/9
Fan et al., 2022	★★★★	★★	★★★	9/9
Thygesen et al., 2020	★★★★	★★	★★★	9/9
Min and Min, 2017	★★★	★★	★★★	8/9
Siddique et al., 2011	★★★	★★	★★	7/9

## 4 Discussion

Our systematic review, which encompasses several studies from diverse regions, provides evidence for the association between prenatal and postnatal exposure to air pollution and the clinical diagnosis of ADHD in children. We have focused our study specifically on subjects clinically diagnosed with ADHD, rather than on children merely exhibiting ADHD-like behaviors or symptoms. This emphasis is critical, as blending clinically diagnosed cases with symptomatic occurrences can lead to confusion and potentially skewed results. Our research aims to further elucidate the relationship between prenatal and postnatal air pollution exposure and the clinical diagnosis of ADHD. This association’s nature and extent illuminate the intricate interplay between environmental factors and neurodevelopmental disorders, underscoring the profound influences that early-life experiences can exert on children’s neurodevelopmental health.

### 4.1 Overview of findings

The pioneering three studies conducted during prenatal period (22–24) have helped to shed light on the intricate relationship between prenatal exposure to specific air pollutants such as PM_2.5_, PM_10_, and NO_x_, and the subsequent development of ADHD. Despite some apparent discrepancies, these findings underscore the importance of further detailed exploration of these associations. Within this body of research, both PM_2.5_ and PM_10_ exposures were evaluated for their potential links to ADHD risk, though the outcomes of these investigations were not entirely consistent. Chang et al. (22) demonstrated a significant correlation between the risk of ADHD and exposure to PM_2.5_ during the first trimester of pregnancy. In contrast, Shih et al. (23) did not identify a significant association between prenatal exposure to PM_10_ and ADHD. These findings imply the possibility of differential health impacts between these two types of particulate matter, warranting additional research to fully discern their respective effects. Moreover, when considering NO_x_ exposure, the studies by Shih et al. (23) and Oudin et al. (24) yielded divergent conclusions. Shih et al. uncovered a clear association between prenatal NO_x_ exposure and an increased risk of hyperactivity disorders. However, Oudin et al. identified a correlation between elevated prenatal NO_x_ exposure and ASD, but not ADHD, in a Swedish population. This suggests that the relationship between NO_x_ exposure and ADHD may be more complex than initially thought, possibly influenced by factors such as geographic location or population genetics. Importantly, Shih et al. reported a stronger correlation between air pollution exposure and ADHD development in boys compared to girls. This finding introduces an additional layer of complexity into these associations, suggesting the possibility of gender-specific responses to environmental exposures, likely influenced by both environmental and biological factors.

Based on the studies we have reviewed, there is an expanding body of evidence indicating an association between postnatal exposure to specific air pollutants, particularly PM_2.5_, PM_10_, and NO_2_, and the risk of developing ADHD in children. Yuchi et al. (25) demonstrated a significant association between exposure to PM_2.5_ during the first 3 years of life and an increased risk of ADHD, thereby emphasizing the potential neurodevelopmental harm of early-life exposure to air pollutants. These findings are consistent with those of Fan et al. (26), who reported an increased risk of ADHD in children exposed to PM_2.5_ and PM_10_. Their large cohort study revealed a notable dose–response relationship, particularly in the upper quartiles of PM_2.5_ and PM_10_ exposure, reinforcing the detrimental effects of particulate matter on neurodevelopment. The role of NO_2_ exposure is further highlighted by the research conducted by Thygesen et al. (30) and Min and Min (28), both of whom observed an increased risk of ADHD in children exposed to NO_2_. Their research presented a complex, potentially dose-dependent, relationship between NO_2_ levels and ADHD, further emphasizing the intricacies of this association.

### 4.2 Methodological considerations

A critical analysis of the methodological approach of the studies included in our review presents opportunities for understanding the relative strengths and potential limitations inherent to the research designs.

The studies in our review employed diverse methodologies, including cohort and cross-sectional designs, which are commonly used in epidemiological research. Cohort studies, as utilized by Chang et al. (22), Shih et al. (23), and Thygesen et al. (27), among others, offer robust evidence due to their prospective nature and the ability to ascertain the temporal sequence between exposure and outcome. However, these designs may also pose challenges due to potential loss to follow-up, resulting in bias. Cross-sectional studies, like those conducted by Forns et al. (21) and Siddique et al. (29), provide insights into prevalent cases and allow the simultaneous measurement of exposure and outcome. However, they inherently lack the ability to establish causal relationships, due to the inability to affirm the temporal sequence of exposure and outcome.

For the exposure assessment, various studies employed environmental databases, air monitoring stations, and satellite-based prediction models, providing a broad spectrum of pollutant exposure measures. While these measures provide reasonably accurate estimates, the possibility of misclassification cannot be completely ruled out, potentially attenuating the true association between air pollutants and ADHD. In the study by Chang et al. (22), for instance, they used high-resolution satellite data for assessing PM_2.5_ exposure. This model-based approach allows for detailed spatial and temporal variation, which can offer more precise exposure estimation. However, it does not consider indoor pollution or personal exposure variability due to lifestyle factors (e.g., time spent outdoors, use of air conditioning), potentially leading to exposure misclassification. Several studies, such as those by Forns et al. (21) and Siddique et al. (29), employed ground monitoring stations for exposure assessment. While these stations can provide accurate measurements, their representation of personal exposure can be limited due to the spatial resolution, particularly in regions with a high degree of spatial variability in air pollution.

In terms of participant age inclusion in the study, our analysis unveiled a diverse age spectrum, spanning from infancy to late adolescence (0–18 years), across the varied studies. Notably, the diagnosis of ADHD frequently emerges during preschool years or adolescence. This disorder’s hallmark is the dynamic progression of symptoms, dependent on age, which adds another layer of complexity to the research paradigm. Consequently, each study enrolled ADHD children at varying developmental stages, which potentially accounts for the disparities in results.

While many studies incorporated adjustments for numerous potential confounders, we must remain vigilant to the persistent threat posed by unmeasured or residual confounding. The intricate web of genetic factors could profoundly modify an individual’s vulnerability to the neurodevelopmental effects of air pollution. Socio-economic status, a multifaceted construct encompassing education level, income, and occupational exposures, can significantly influence both the degree of air pollution exposure and access to resources that mitigate its effects. These elements underscore the complexity of disentangling the multifactorial etiology of ADHD, necessitating advanced statistical techniques such as instrumental variable analysis and propensity score matching to robustly account for such confounders. Future research should strive to incorporate comprehensive, longitudinal data collection that can capture these subtleties, thus providing a more precise elucidation of how air pollution interacts with a constellation of genetic, psychological, and socio-economic factors to influence ADHD risk (31).

When evaluating the link between air pollution and ADHD risk, it is critical to consider spatial and temporal exposure variations. Geographically, exposure differs markedly between high and low-income regions, influenced by local industrial and vehicular emissions which vary with economic, regulatory, and technological contexts. Temporally, pollution fluctuates with seasonal traffic, heating, and industrial activities, and evolves with changing environmental policies. Therefore, dynamic exposure assessments using advanced techniques like land use regression models and extensive air quality monitoring networks are essential for accurate evaluation.

In conclusion, while our review builds on the strength of diverse methodologies, accurate exposure assessment, and ADHD diagnosis, the complexity of the association necessitates further rigorous research designs that address the limitations.

### 4.3 Research implications

The complexity and heterogeneity of study methodologies, ranging from the types of pollutants and their measurement methods, covariates, ADHD assessment, diagnostic criteria, to sample sizes, pose a significant challenge in drawing a definitive conclusion. Despite existing research outlining the link between air pollution and various neurodevelopmental disorders, there remains a persistent need for deeper investigation into this topic and its ramifications on children’s health. Future studies exploring the association between prenatal and postnatal air pollution exposure and ADHD must consider refining the diagnostic criteria to alleviate confusion arising from conflating clinically diagnosed ADHD and ADHD-like behaviors. This endeavor necessitates a robust, clinically-informed diagnostic procedure, incorporating multidimensional evaluations from diverse sources such as parents, teachers, and clinicians. Furthermore, it is critical to recognize the potential influence of the varying effects of air pollution across different developmental stages. By differentiating between prenatal and postnatal exposure periods, we may uncover nuanced impacts, thereby enhancing our understanding of ADHD etiology (15). While existing literature primarily underscores the effects of PM_2.5_ and NO_x_, the impacts of other air pollutants have been relatively overlooked (32). Hence, expanding research focus to include other forms of pollutants could pave the way to a more comprehensive understanding of air pollution’s influence on ADHD risk. Additionally, it could further explore innovative approaches to address these challenges, such as the use of personal monitoring devices for more accurate exposure measurement or longitudinal designs to better establish temporality and causality. Moreover, our review underscores the urgent need for further exploration into the relatively uncharted moderating factors, such as socio-economic status, genetic predispositions, and the role of psychosocial stressors in the air pollution-ADHD relationship. Our review also underscores the inconsistencies in the geographical distribution of studies, with a significant majority conducted in high-income countries. Given the diversity in air quality and ADHD diagnostic practices across regions, it is imperative for future research to incorporate a wider array of settings. In particular, regions with substandard air quality, like some low-and-middle-income countries, may reveal more pronounced effects (33).

### 4.4 Limitations of the review

Our analysis encountered certain limitations. First, the potential for publication bias. Studies yielding null or negative results may not attain the same publication frequency as those reporting positive associations, potentially exaggerating the link between prenatal and postnatal air pollution exposure and ADHD. Despite our comprehensive search strategies, this bias remains a pervasive issue. Secondly, the inherent limitations of observational studies compromise the concern of unmeasured or residual confounding factors influencing our analysis. Despite the high NOS scores of our selected studies, residual confounding remains a relevant challenge after careful design and attempts to control confounding factors. And the use of the NOS for quality assessment in studies introduces inherent limitations due to the subjective interpretation of its criteria. This subjectivity can lead to scoring variability among reviewers. Despite efforts to mitigate these issues through a dual-review process and resolving discrepancies by discussion, some degree of subjectivity and bias remains unavoidable. Thirdly, diverse methodologies across the studies evaluating air pollution exposure and diagnosing ADHD add another layer of complexity. While some studies relied on area-level exposure assessments, others used personal exposure measurements. This divergence could contribute to the heterogeneity of our findings and restrict direct comparisons across studies. Moreover, variability in diagnostic criteria and sources for ADHD diagnosis further complicates our findings’ interpretation. Lastly, we restricted our review to articles published in English, which may have excluded significant research from regions where non-English languages predominate. This limitation could potentially restrict the generalizability of our findings across various geographic and cultural contexts. Despite these hurdles, this systematic review provides valuable insights into the potential link between prenatal and postnatal air pollution exposure and ADHD. Future research should address these limitations to elucidate this intricate relationship further.

## 5 Conclusion

The present systematic review meticulously synthesizes data from eight studies, each focusing on the impact of prenatal and postnatal exposure to air pollutants on the development of ADHD in children. Notably, the prenatal studies revealed a significant correlation between the risk of ADHD and exposure to PM_2.5_, but interestingly, found no such association with PM_10_. As for the prenatal exposure to NO_x_, the findings were notably diverse, with one study linking prenatal NO_x_ exposure to a higher risk of ADHD, while another found it associated with ASD, not ADHD. On the other hand, most studies reported a connection between ADHD and postnatal air pollution exposure, thereby highlighting an expanding body of evidence suggesting an association between postnatal exposure to specific air pollutants, particularly PM_2.5_, PM_10_, and NO_2_, and the risk of developing ADHD in children. Although epidemiological studies suggest that air pollutants can contribute to ADHD, the data on prenatal and postnatal exposure still must be thoroughly explored due to the heterogeneity observed in the odd ratios forest plot and the methodological discrepancies identified across the studies. Even despite the high NOS scores of our selected studies, it is recommended to interpret the results with caution. Future studies should aim to minimize heterogeneity and reduce the risk of bias by ensuring a more representative sample, standardizing exposure, and outcome assessments. In the light of these findings, further research needs to be conducted on this topic. Specifically, consideration should be given to each pollutant, and methodologies should be kept consistent to better understand the effect of air pollution exposure on the development of ADHD. Furthermore, these findings underscore the need for public health policy interventions aimed at reducing air pollution exposure, which could potentially mitigate the risk of ADHD and other developmental disorders in children.

## Author contributions

JZ: Writing – original draft, Software, Formal analysis, Data curation. TH: Writing – original draft. FW: Formal analysis, Data curation, Writing – original draft. WL: Writing – review & editing, Methodology.
