# Supplementary material for: Association of prenatal and postnatal exposure to air pollution with clinically diagnosed attention deficit hyperactivity disorder: a systematic review
Source: Front Public Health. 2024 May 24;12:1396251. doi: 10.3389/fpubh.2024.1396251 (PMC11157082; doi:10.3389/fpubh.2024.1396251)
Supplement: Supplementary file 2 [file Table_2.docx]

**Appendix 2**. Detailed search strategy

**Pubmed**

**("air pollution"[MH]** OR "air pollution"[TW] OR "outdoor pollution"[TW] OR "air quality"[TW] OR "polluted air"[TW] OR "air quality"[TW] OR "contaminated air"[TW] OR "air pollutions"[TW] OR "pollution, air"[TW] OR "Air Pollutant"[TW] OR "Air Pollutants"[TW] OR "Airborne Pollutants"[TW] OR "Air Toxics"[TW] OR "Airborne Pollutant"[TW] OR "Air Toxic"[TW])

**AND**

(**"attention deficit disorder with hyperactivity"[MH]** OR "attention deficit disorder with hyperactivity"[TW] OR "attention deficit disorders with hyperactivity"[TW] OR "ADHD"[TW] OR "attention deficit hyperactivity disorder"[TW] OR "hyperkinetic syndrome"[TW] OR "syndromes, hyperkinetic"[TW] OR "attention deficit-hyperactivity disorder"[TW] OR "attention deficit-hyperactivity disorders"[TW] OR "deficit-hyperactivity disorder, attention"[TW] OR "deficit-hyperactivity disorders, attention"[TW] OR "disorder, attention deficit-hyperactivity"[TW] OR "disorders, attention deficit-hyperactivity"[TW] OR "ADDH"[TW] OR "attention deficit hyperactivity disorders"[TW] OR "attention deficit disorder"[TW] OR "attention deficit disorders"[TW] OR "deficit disorder, attention"[TW] OR "deficit disorders, attention"[TW] OR "disorder, attention deficit"[TW] OR "disorders, attention deficit"[TW] OR "brain dysfunction, minimal"[TW] OR "dysfunction, minimal brain"[TW] OR "minimal brain dysfunction" [TW])

**Embase 236**

('air pollutant'/exp OR ‘air pollution’ OR ‘outdoor pollution’ OR ‘air quality’ OR ‘polluted air’ OR ‘air quality’ OR ‘contaminated air’ OR ‘air pollutions’ OR ‘pollution, air’ OR ‘Air Pollutant’ OR ‘Air Pollutants’ OR ‘Airborne Pollutants’ OR ‘Air Toxics’ OR ‘Airborne Pollutant’ OR ‘Air Toxic’)

AND

('attention deficit hyperactivity disorder'/exp OR 'attention deficit hyperactivity disorder' OR 'attention deficit disorder with hyperactivity' OR 'attention deficit disorders with hyperactivity' OR 'ADHD' OR 'attention deficit hyperactivity disorder' OR 'hyperkinetic syndrome' OR 'syndromes, hyperkinetic' OR 'attention deficit-hyperactivity disorder' OR 'attention deficit-hyperactivity disorders' OR 'deficit-hyperactivity disorder, attention' OR 'deficit-hyperactivity disorders, attention' OR 'disorder, attention deficit-hyperactivity' OR 'disorders, attention deficit-hyperactivity' OR 'ADDH' OR 'attention deficit hyperactivity disorders' OR 'attention deficit disorder' OR 'attention deficit disorders' OR 'deficit disorder, attention' OR 'deficit disorde')

**Web of Science 402**

**TS=(**outdoor pollution OR air pollution OR polluted air OR air quality OR contaminated air OR air pollutions OR pollution, air OR Air Pollutant OR Air Pollutants OR Airborne Pollutants OR Air Toxics OR Airborne Pollutant OR Air Toxic)

AND

**TS=**(attention deficit disorder with hyperactivity OR attention deficit disorders with hyperactivity OR ADHD OR attention deficit hyperactivity disorder OR hyperkinetic syndrome OR syndromes, hyperkinetic OR attention deficit-hyperactivity disorder OR attention deficit-hyperactivity disorders OR disorder, attention deficit-hyperactivity OR ADDH OR attention deficit hyperactivity disorders OR attention deficit disorder OR attention deficit disorders OR minimal brain dysfunction)

**(**‘air pollution’ OR ‘outdoor pollution’ OR ‘air quality’ OR ‘polluted air’ OR ‘air quality’ OR ‘contaminated air’ OR ‘air pollutions’ OR ‘pollution, air’ OR ‘Air Pollutant’ OR ‘Air Pollutants’ OR ‘Airborne Pollutants’ OR ‘Air Toxics’ OR ‘Airborne Pollutant’ OR ‘Air Toxic’)
