# Supplementary material for: Association of prenatal and postnatal exposure to air pollution with clinically diagnosed attention deficit hyperactivity disorder: a systematic review
Source: Front Public Health. 2024 May 24;12:1396251. doi: 10.3389/fpubh.2024.1396251 (PMC11157082; doi:10.3389/fpubh.2024.1396251)
Supplement: Supplementary file 3 [file Table_3.docx]

**Table S1. Data Extracted from Articles on Prenatal and Postnatal Air Pollutant Exposure and ADHD like behavior**

| Author & year | Country | Study desigh | Exposure period | Study pollution | Age group | Total (n) | Exposure (Air pollutants) | How exposure measured | Test | Adjustment of risk | Main Findings |
| --- | --- | --- | --- | --- | --- | --- | --- | --- | --- | --- | --- |
| Li et al. 2023(Li et al. 2023) | Netherlands | Cohort | Postnatal | Children from the TRacking Adolescents’ Individual Lives Survey (TRAILS). | 10-12 years old | 2,750 | O_3_  SO_2_  NO_2_  PM_2.5_  PM_10_ | Land Use Regression model (LUR) | Child Behavior Checklist and the Adult Behavior Checklist  (CBCL) | Socioeconomic status, children’s use of psychostimulant medication, the interaction between follow-up time and sex, the interaction between follow-up time and children’s use of psychostimulant medication, problematic pregnancy or child delivery, maternal smoking during pregnancy, mothers’ age at child delivery, pregnancy duration, and lifetime parental psychopathology. | Higher levels of exposure to PM were associated with more severe ASD and ADHD symptoms. |
| Choi et al. 2023(Choi et al. 2023) | Korea | Cohrot | Prenatal and Postnatal period | Children form Environment and Development of Children (EDC) Study | 4-8 years old | 329 | SO_2_ | The nearest  monitor approach | ADHD rating scale (ARS) | Maternal age at pregnancy, maternal educational level, maternal smoking status during pregnancy, ETS during pregnancy, child’s sex, gestational age, multiple births, season of child’s birth, cell type fraction, and maternal IQ evaluated by the short version of the Korean Wechsler Adult Intelligence Scale | Prenatal SO2 exposure was associated with ADHD symptoms. |
| Author & year | **Country** | **Study desigh** | **Exposure period** | **Study pollution** | **Age group** | **Total (n)** | **Exposure (Air pollutants)** | **How exposure measured** | **Test** | **Adjustment of risk** | **Main Findings** |
| Liu et al. 2022 (Liu et al. 2022) | Chian  (Shenzhen) | Cohort | Prenatal period | Children from Longhua Child Cohort Study (LCCS) | 3-17 years | 26,052 | SO_2_  NO_2_  CO O_3_  PM_2.5_ PM_10_ | Land-use random forest model (LURF) | Conners’ Parent Rating Scale | Sex, age, average daily sleep duration, feeding pattern, and birth weight, maternal age at birth, delivery way, parity, maternal passive smoking or alcohol consumption during pregnancy, multivitamins, folic acid or calcium supplementation during pregnancy, and gestational diseases, family/environment | An association was found between NO_2_, PM_10_, PM_2.5_ and hyperactivity |
| Forns et al. 2016 (Forns et al. 2016) | Spain  (Barcelona) | Cross sectional | Postnatal period | Children form the BRain dEvelopment and Air polluTion ultrafine particles in scHool childrEn (BREATHE) project | 7-11 years | 2,720 | EC  BC  NO_2_ | Temporally adjusted land-use regression (LUR)  models | Parental report  SDQ Teachers reported the ADHD Criteria  (DSM-IV) list | Child’s sex and age maternall education urban vulnerability index at home  air pollution (BC) at home home tobacco use urban vulnerability index at school type of school noise annoyance at home siblings at birth duration of breast feeding. | The positive association between exposure to outdoor EC, BC and NO_2_ with SDQ score  And no significant association was found for EC, BC and NO2 with ADHD symptoms scores |
| Author & year | **Country** | **Study desigh** | **Exposure period** | **Study pollution** | **Age group** | **Total (n)** | **Exposure (Air pollutants)** | **How exposure measured** | **Test** | **Adjustment of risk** | **Main Findings** |
| Peterson et al. 2022 (Peterson et al. 2022) | USA | Cohort | Prenatal period | Pregnant African–American or Dominican women residents of northern Manhattan recruited through local prenatal clinics | 6-14 years | 332 | PM_2.5_ | PM_2.5_ exposure measured  for each day of pregnancy using spatiotemporal exposure models at home addresses | ADHD severity Rating Scale. | Sex, child handedness, maternal ethnicity and maternal education, material hardship and quality of home environment | PM_2.5_ exposure were found not significantly associated with any of the behavioural outcomes, ADHD severity or anxiety severity |
| Maitre et al. 2021 (Maitre et al. 2021) | BiB in United Kingdom, EDEN in France, KANC in  Lithuania, INMA in Spain, MoBa in Norway, and RHEA in Greece | Cohort | Prenatal and Postnatal period | Children from the HELIX (Human Early Life Exposome) project | 6-11 years | 1,301 | NO_2_  PM_2.5_  PM_10_  PM_abs_ | Remote sensing  and geo-spatial methods | Conner rating scale and child behavior  checklist (CBCL) | Cohort, maternal age, child age, child gender, season of  conception and maternal education | Indoor air pollution, unhealthy diet were associated with increased scores |
| McGuinn et al. 2020 (McGuinn et al. 2020) | Mexico | Cohort | Prenatal period | Children from the Programming Research in Obesity, Growth, Environment and Social Stressors (PROGRESS) study | 4-6 years | 539 | PM_2.5_ | 1 km based satellite-based estimation model | Behavioral Assessment System for Children （BASC-2） | Sex, age, maternal age at enrolment, years of education, depressive  symptoms during pregnancy, and  maternal IQ, SES, home caretaking environment, and season of conception | An association has been observed between prenatal exposure to PM_2.5_ during the first trimester and an increase in scores across various behavioral subscales |
| Author & year | **Country** | **Study desigh** | **Exposure period** | **Study pollution** | **Age group** | **Total (n)** | **Exposure (Air pollutants)** | **How exposure measured** | **Test** | **Adjustment of risk** | **Main Findings** |
| Mortamais et al. 2019 (Mortamais et al. 2019) | Barcelona, Catalonia, Spain | Cohort | Prenatal period | Children from Brain Development and Air Pollution Ultrafine Particles in School Children (BREATHE) project | 8-12 years | 181 | PM_2.5_ | Land use regression models (LUR) | Strengths and Difficulties Questionnaire (SDQ) and the criteria of the  Attention Deficit Hyperactivity Disorder DSM-IV list | Age at MRI, sex, intracranial volume, maternal education, residential neighborhood socioeconomic status,  birthweight and mother's smoking status during pregnancy | Prenatal exposure to PM_2.5_, particularly during the last trimester of pregnancy, may induce structural changes in the corpus callosum in children aged between 8 and 12 years from the general population |
| Roberts et al. 2019 (Roberts et al. 2019) | England and Wales | Cohort | Postnatal period | Children from the Environmental Risk (E-Risk) Longitudinal Twin study | 7-18 years | 284 | PM_2.5_  NO_2_ | KCLurban model | ADHD symptoms at age 12 were ascertained via by others‘ and teachers‘ reports of inattention and hyperactivity-impulsivity according to DSM-IV criteria and the Rutter Child Scales. | sex, ethnicity, neighborhood SES, family SES, family psychiatric history, exposure to severe victimization, and smoking | No associations between age-12 pollution exposure and concurrent mental health problems |
| Sentís et al. 2019 (Sentís et al. 2017) | Spanish | Cohort | Prenatal and Postnatal period | Children from the Spanish INMA—Environment and Childhood—Project | 4-5 years | 1,298 | NO_2_ | Land-use regression models (LUR) | Kiddie-Conners Continuous Performance Test (K-CPT) | Child's sex and age at the time of the attention function test, maternal and paternal educational level, maternal and  paternal age, parents' social class, maternal and paternal countries of birth et al. | Higher exposure to ambient NO_2_, mainly during pregnancy and to a lesser extent postnatal, is associated with impaired attention function in children at 4–5 years of age. |
| Author & year | **Country** | **Study desigh** | **Exposure period** | **Study pollution** | **Age group** | **Total (n)** | **Exposure (Air pollutants)** | **How exposure measured** | **Test** | **Adjustment of risk** | **Main Findings** |
| Forns et al. 2018 (Forns et al. 2018) | Denmark  Netherlands  Germany  France  Italy  Spain  Sweden | Cohort | Prenatal period | Children from Eropean Study of Cohorts for Air Pollution Effects (ESCAPE) Project | 3-10 years | 29,127 | PM_10_  PM_2.5_  PM coarse  NO_2_ | Land use regression models (LUR) | A-TAC,  the Child Behavior Checklist for Toddlers (CBCL), the Strengths and Difficulties  Questionnaire (SDQ), and the ADHD Criteria of Diagnostic and Statistical Manual of Mental Disorders, Fourth Edition  (ADHD-DSM-IV) list | Maternal education or socioeconomic level, country of birth, maternal age at delivery, pre-pregnancy body mass index, height, prenatal smoking, parity, child’s sex, season at child’s birth, type of zone at child’s birth address, child’s age at assessment, type of evaluator of the test | No association between exposures and ADHD symptoms scores |
| Alemany et al. 2018 (Alemany et al. 2018) | European | Cohort | Postnatal period | Children from the BREATHE project | 7-11 years | 2,897 | PAHs  EC  NO_2_ | Outdoor PAHs, EC, and NO_2_ measured at each school as surrogates for TRAP exposure at each school | Strengths and Difficulties Questionnaire (SDQ), computerized tests assessing inattentiveness and working memory | Gender, age at baseline, maternal education, and residential neighborhood SES | Children carrying the APOE e4 allele than in other children, which suggests that e4 carriers may be more vulnerable to adverse neurobiological effects of TRAP exposure than noncarriers. |
| Author & year | **Country** | **Study desigh** | **Exposure period** | **Study pollution** | **Age group** | **Total (n)** | **Exposure (Air pollutants)** | **How exposure measured** | **Test** | **Adjustment of risk** | **Main Findings** |
| Sentís et al. 2017 (Sentís et al. 2017) | Spanish  (Valencia, Sabadell, Asturias, Gipuzkoa) | Cohort | Prenatal and postnatal period | Children from the INfanciay Medio Ambiente (INMA) Project | 4-5 years | 1298 | NO_2_ | Land-use regression models (LUR) | The 2nd edition of the Conners Kiddie Continuous  Performance Test (K-CPT) | Maternal and parental education, age, parents' social class, countries of birth, height and pre-pregnancy weight, paternal BMI , maternal smoking and exposure to second-hand smoke, maternal alcohol use, maternal consumption of fish, fruit, vegetables, vitamin D, and folic acid, maternal noise annoyance, household gas appliances, child's sex, number of siblings, maternal mental health, maternal verbal IQ, child age at the time of the test, residence urbanicity during pregnancy | Prenatal NO_2_ exposure was associated with an impaired standard error of the hit reaction time and increased omission |
| Sunyer et al. 2017 (Sunyer 2017) | Spain  (Barcelona) | Cohort | Postnatal period | Children form 39 schools in Barcelona | 7-10 years | 2715 | NO_2_  EC | Fixed air quality background  monitoring station | Attentional  network test (ANT, computerized  tests) | Temperature and relative, humidity on the current day, season , day of the week and period, hour of exam, child’s age, sex, maternal education, socioeconomic status of the neighborhood of residence, home air pollution | Both NO_2_ and EC had associations with inattentiveness |
| Author & year | **Country** | **Study desigh** | **Exposure period** | **Study pollution** | **Age group** | **Total (n)** | **Exposure (Air pollutants)** | **How exposure measured** | **Test** | **Adjustment of risk** | **Main Findings** |
| Yorifuji  et al.  2017 (Yorifuji et al. 2017) | Japan | Cohort | Prenatal period | Children from the Longitudinal Survey of Babies in the 21st Century | 8 years | Interrupting 25,677  Inability to wait his/her turn during play  25,797  Failure to pay attention when crossing a street 25,773 | PM < 7 µm NO_2_  SO_2_ | Monitoring stations in each municipality | Child Behavior  Checklist (CBCL).  Three questions were related to  attention problems: 1) Does your child interrupt people? 2) Can your child wait for his/her turn during play? 3) Can your child pay attention  to the surrounding area when  crossing the street? | Sex, birth month,  Parity, maternal age at delivery, maternal smoking habits, maternal educational level, paternal income during the year in which the child was born, type of municipality in which participants were born  per capita taxable income population density of each municipality | Prenatal exposure to air pollution was positively associated with risk for behavioral problems related to attention |
| Chiu et al. 2016 (Chiu et al. 2016) | The U.S  (Boston) | Cohort | Prenatal period | Children from Asthma Coalition on Community, Environment  and Social Stress (ACCESS) project | 6-7 years | 267 | PM_2.5_ | Validated satellite based spatiotemporally resolved prediction model. (MODIS and LUR) | Conners’  Continuous  Performance TestII (CPT-II) | Maternal age, race, education, prenatal/postnatal, maternal smoking, parity, blood lead level at neurodevelopmental  Testing, Child sex | No significant associations were found |
| Author & year | **Country** | **Study desigh** | **Exposure period** | **Study pollution** | **Age group** | **Total (n)** | **Exposure (Air pollutants)** | **How exposure measured** | **Test** | **Adjustment of risk** | **Main Findings** |
| Fuertes et al. 2016 (Fuertes et al. 2016) | German | Cohort | Postnatal period | Children from the “German Infant study on the influence of a Nutritional Intervention plus environmental and genetic influences on allergy development” (GINIplus) and “Lifestyle-related factors, Immune  System and the development of Allergies in East and West Germany  plus the influence of traffic emissions and genetics study” (LISAplus) | 10-15 years | 4,745 | NO_2_  PM_10_  PM_2.5_ | Land use regression models (LUR) | German parent-completed (at age 10 years) and self-completed (at age 15 years) versions of the SDQ | Sex, exact age at follow up, parental education, maternal age at birth, maternal smoking, child secondhand smoking  Time spent in front of a screen, time spent outside, single parent status, parental psychology, population density, distance to green spaces | Hyperactivity and inattention symptoms at the ages of 10 and 15 years were found to be associated with both PM_2.5_ mass and estimated absorbance. |
| Hjortebjerg  et al. 2016 (Hjortebjerg et al. 2016) | Denmark | Cohort | Postnatal period | Children from the population-based Danish National Birth Cohort (DNBC) | 7 years | 46,940 | NO_x_ | Danish AirGIS  modeling system  (based on the  Operational  Street Pollution Model (OSPM) | Parental reported Danish parent reported version of the SDQ | Sex, age at SDQ, gestational age, birth weight, maternal age at delivery, parity, smoking and alcohol consumption, level of education, disposable income, railway & airport noise (at birth & SDQ), maternal mental health | NO_x_ did not display any association with behavioral problems. |
| Kicinski et al.  2016 (Kicinski et al. 2016) | Belgium | Cross- sectional | Postnatal period | Children from the biomonitoring program for en- vironmental health surveillance in Flanders, Belgium | 14-15  years | 832 | NO_2_ | Spatial-temporal interpolation  method | Continuous  Performance Test  (CPT) | Sex, age, passive smoking, education of parents, ethnicity, urinary creatinine, day of the week, time of day, lood lead | A significant association between an increase in Urinary t,t-muconic acid and sustained attention. |
| Author & year | **Country** | **Study desigh** | **Exposure period** | **Study pollution** | **Age group** | **Total (n)** | **Exposure (Air pollutants)** | **How exposure measured** | **Test** | **Adjustment of risk** | **Main Findings** |
| Yorifuji et al.  2016 (Yorifuji et al. 2016) | Japan | Cohort | Prenatal period | Children from the s the Longitudinal Survey of Babies in the 21st Century | 5.5 years | 46,039 | PM < 7 µm NO_2_  SO_2_ | Monitoring stations throughout Japan | Some questions  were asked on the nationwide survey (parental  complete attention questions) | Sex, birth month, maternal age at delivery, parity, maternal smoking status, maternal educational level, paternal income, municipality-level variables: residential area, per capita, income, population density | At 5.5 years of age, there was an observed association between air pollution and behaviors linked to inhibition and impulsivity |
| Sunyer et al. 2015 (Sunyer et al. 2015) | Spain | Cohort | Postnatal period | Children from the forty schools in Barcelona (Catalonia, Spain) w | 7-10 years | 2,715 | EC  NO_2_ | The average of the two 1-week measuring adjusted for temporal | Attentional Network Test (ANT, computerized tests) | Age, sex, maternal education, residential neighborhood, socioeconomic status, air pollution exposure at home, school | Children exposed to higher levels of traffic-related air pollutants at school. |
| Gong et al. 2014 (Gong et al. 2014) | Swedish (Stockholm) | Cohort study | Prenatal period | Children from the Child and Adolescent Twin Study in Sweden (CATSS) Project | 9-12 years | 3,426 | PM_10_ NO_x_ | Dispersion modeling controlled for seasonal variation | Parental reported (A-TAC based on DSM-IV criteria) | Parity, gender, maternal age during pregnancy, maternal marital status, parental education, Family income, neighborhood deprivation index at birth year, maternal smoking during pregnacy | No association between exposures and ADHD |
| Author & year | **Country** | **Study desigh** | **Exposure period** | **Study pollution** | **Age group** | **Total (n)** | **Exposure (Air pollutants)** | **How exposure measured** | **Test** | **Adjustment of risk** | **Main Findings** |
| Van kempen et al. 2012 (van Kempen et al. 2012) | The Netherlands (Amsterdam) | Cross-sectional | Postnatal period | Children from 24 primary schools around Schiphol-Amsterdam Airport. | 9-11 years | 485 | NO_2_ | Models on the basis of monitored data in routine measurement sites, land use and topography. | The Neurobehavioral Evaluation System (NES): -The Switching Attention Test (SAT)  - The Simple Reaction Time Test (SRTT) | Age, gender, employment status, crowding, home ownership, mother’s education, long-standing illness, the main language spoken, at home, parental support, type of window glazing at school, road traffic noise, aircraft noise exposure | No associations were identified between school-based NO2 exposure and reaction speed, as well as switching attention both at school and home. |
| Morales, E.  2009 (Morales et al. 2009) | Spain | Cohort | Prenatal period | Children from a population-based birth cohort recruited on the island of Menorca (Spain) | 4 years | 398 | NO_2_ | Average 2-week NO_2_ concentrations were assessed using passive diffusion tubes placed on the living room wall at a height of 2 meters | The ADHD Diagnostic and Statistical Manual of Mental Disorders, 4th  Edition questionnaire | Maternal social class, maternal education, school trimester at  testing, evaluator (neuropsychologist), maternal smoking during pregnancy, number of smokers at home, maternal  alcohol consumption during pregnancy, and home location | Early-life exposure to air pollution from indoor gas appliances may be negatively associated with neuropsychological development through the first 4 years of life. |
| Wang et al. 2009 (Wang et al. 2009) | China  (Quanzhou) | Cross-sectional | Postnatal period | Children from second grade (8–9 years of age) and third grade (9–10 years of age) from two schools in Quanzhou | 8-10 years | 133 | NO_2_  PM_10_ | Monitoring sites in each primary school  (by passive samplers) | Continuous  Performance (CPT) | Age, BMI, educational attainment of subjects’ father, exposure status (clear or polluted), sex, birth weight, delivery method, second-hand smoke, open kitchen, household fuel, familiarity with computer, games, vision, breastfeeding | Significant associations were found between traffic air pollution exposure and poorer performance on CPT neurobehavioral tests. |

**References**

Alemany, S., N. Vilor-Tejedor, R. García-Esteban, M. Bustamante, P. Dadvand, M. Esnaola, M. Mortamais, J. Forns, B. L. van Drooge, M. Álvarez-Pedrerol, J. O. Grimalt, I. Rivas, X. Querol, J. Pujol & J. Sunyer (2018) Traffic-related air pollution, APOE ∊4 status, and neurodevelopmental outcomes among school children enrolled in the BREATHE project (Catalonia, spain). *Environmental Health Perspectives,* 126.

Chiu, Y. H., H. H. Hsu, B. A. Coull, D. C. Bellinger, I. Kloog, J. Schwartz, R. O. Wright & R. J. Wright (2016) Prenatal particulate air pollution and neurodevelopment in urban children: Examining sensitive windows and sex-specific associations. *Environ Int,* 87**,** 56-65.

Choi, Y.-J., J. Cho, Y.-C. Hong, D.-W. Lee, S. Moon, S. J. Park, K.-S. Lee, C. H. Shin, Y. A. Lee, B.-N. Kim, Z. Kaminsky, J. I. Kim & Y.-H. Lim (2023) DNA methylation is associated with prenatal exposure to sulfur dioxide and childhood attention-deficit hyperactivity disorder symptoms. *Scientific reports,* 13**,** 3501-3501.

Forns, J., P. Dadvand, M. Foraster, M. Alvarez-Pedrerol, I. Rivas, M. López-Vicente, E. Suades-Gonzalez, R. Garcia-Esteban, M. Esnaola, M. Cirach, J. Grellier, X. Basagaña, X. Querol, M. Guxens, M. J. Nieuwenhuijsen & J. Sunyer (2016) Traffic-Related air pollution, noise at school, and behavioral problems in barcelona schoolchildren: A cross-sectional study. *Environmental Health Perspectives,* 124**,** 529-535.

Forns, J., J. Sunyer, R. Garcia-Esteban, D. Porta, A. Ghassabian, L. Giorgis-Allemand, T. Gong, U. Gehring, M. Sorensen, M. Standl, D. Sugiri, C. Almqvist, A. Andiarena, C. Badaloni, R. Beelen, D. Berdel, G. Cesaroni, M.-A. Charles, K. T. Eriksen, M. Estarlich, M. F. Fernandez, A. Forhan, V. W. V. Jaddoe, M. Korek, P. Lichtenstein, A. Lertxundi, M.-J. Lopez-Espinosa, I. Markevych, A. de Nazelle, O. Raaschou-Nielsen, M. Nieuwenhuijsen, R. Perez-Lobato, C. Philippat, R. Slama, C. M. T. Tiesler, F. C. Verhulst, A. von Berg, T. Vrijkotte, A.-M. N. Andersen, B. Heude, U. Kramer, J. Heinrich, H. Tiemeier, F. Forastiere, G. Pershagen, B. Brunekreef & M. Guxens (2018) Air Pollution Exposure During Pregnancy and Symptoms of Attention Deficit and Hyperactivity Disorder in Children in Europe. *Epidemiology,* 29**,** 618-626.

Fuertes, E., M. Standl, J. Forns, D. Berdel, J. Garcia-Aymerich, I. Markevych, G. Schulte-Koerne, D. Sugiri, T. Schikowski, C. M. Tiesler & J. Heinrich (2016) Traffic-related air pollution and hyperactivity/inattention, dyslexia and dyscalculia in adolescents of the German GINIplus and LISAplus birth cohorts. *Environ Int,* 97**,** 85-92.

Gong, T., C. Almqvist, S. Bölte, P. Lichtenstein, H. Anckarsäter, T. Lind, C. Lundholm & G. Pershagen (2014) Exposure to air pollution from traffic and neurodevelopmental disorders in Swedish twins. *Twin Res Hum Genet,* 17**,** 553-62.

Hjortebjerg, D., A. M. Andersen, J. S. Christensen, M. Ketzel, O. Raaschou-Nielsen, J. Sunyer, J. Julvez, J. Forns & M. Sørensen (2016) Exposure to Road Traffic Noise and Behavioral Problems in 7-Year-Old Children: A Cohort Study. *Environ Health Perspect,* 124**,** 228-34.

Kicinski, M., N. D. Saenen, M. K. Viaene, E. Den Hond, G. Schoeters, M. Plusquin, V. Nelen, L. Bruckers, I. Sioen & I. Loots (2016) Urinary t,t-muconic acid as a proxy-biomarker of car exhaust and neurobehavioral performance in 15-year olds. *Environmental Research,* 151**,** 521-527.

Li, Y., T. Xie, R. D. Cardoso Melo, M. de Vries, J. Lakerveld, W. Zijlema & C. A. Hartman (2023) Longitudinal effects of environmental noise and air pollution exposure on autism spectrum disorder and attention-deficit/hyperactivity disorder during adolescence and early adulthood: The TRAILS study. *Environmental Research,* 227.

Liu, B., X. Fang, E. Strodl, G. He, Z. Ruan, X. Wang, L. Liu & W. Chen (2022) Fetal Exposure to Air Pollution in Late Pregnancy Significantly Increases ADHD-Risk Behavior in Early Childhood. *Int J Environ Res Public Health,* 19.

Maitre, L., J. Julvez, M. López-Vicente, C. Warembourg, I. Tamayo-Uria, C. Philippat, K. B. Gützkow, M. Guxens, S. Andrusaityte, X. Basagaña, M. Casas, M. de Castro, L. Chatzi, J. Evandt, J. R. Gonzalez, R. Gražulevičienė, L. Smastuen Haug, B. Heude, C. Hernandez-Ferrer, M. Kampouri, D. Manson, S. Marquez, R. McEachan, M. Nieuwenhuijsen, O. Robinson, R. Slama, C. Thomsen, J. Urquiza, M. Vafeidi, J. Wright & M. Vrijheid (2021) Early-life environmental exposure determinants of child behavior in Europe: A longitudinal, population-based study. *Environment International,* 153.

McGuinn, L. A., D. C. Bellinger, E. Colicino, B. A. Coull, A. C. Just, I. Kloog, E. Osorio-Valencia, L. Schnaas, R. J. Wright, M. M. Téllez-Rojo, R. O. Wright & M. K. Horton (2020) Prenatal PM(2.5) exposure and behavioral development in children from Mexico City. *Neurotoxicology,* 81**,** 109-115.

Morales, E., J. Julvez, M. Torrent, R. de Cid, M. Guxens, M. Bustamante, N. Kuenzli & J. Sunyer (2009) Association of Early-life Exposure to Household Gas Appliances and Indoor Nitrogen Dioxide With Cognition and Attention Behavior in Preschoolers. *American Journal of Epidemiology,* 169**,** 1327-1336.

Mortamais, M., J. Pujol, G. Martínez-Vilavella, R. Fenoll, C. Reynes, R. Sabatier, I. Rivas, J. Forns, N. Vilor-Tejedor, S. Alemany, M. Cirach, M. Alvarez-Pedrerol, M. Nieuwenhuijsen & J. Sunyer (2019) Effects of prenatal exposure to particulate matter air pollution on corpus callosum and behavioral problems in children. *Environ Res,* 178**,** 108734.

Peterson, B. S., R. Bansal, S. Sawardekar, C. Nati, E. R. Elgabalawy, L. A. Hoepner, W. Garcia, X. Hao, A. Margolis, F. Perera & V. Rauh (2022) Prenatal exposure to air pollution is associated with altered brain structure, function, and metabolism in childhood. *Journal of child psychology and psychiatry, and allied disciplines,* 63**,** 1316-1331.

Roberts, S., L. Arseneault, B. Barratt, S. Beevers, A. Danese, C. L. Odgers, T. E. Moffitt, A. Reuben, F. J. Kelly & H. L. Fisher (2019) Exploration of NO2 and PM2.5 air pollution and mental health problems using high-resolution data in London-based children from a UK longitudinal cohort study. *Psychiatry Research,* 272**,** 8-17.

Sentís, A., J. Sunyer, A. Dalmau-Bueno, A. Andiarena, F. Ballester, M. Cirach, M. Estarlich, A. Fernández-Somoano, J. Ibarluzea, C. Íñiguez, A. Lertxundi, A. Tardón, M. Nieuwenhuijsen, M. Vrijheid & M. Guxens (2017) Prenatal and postnatal exposure to NO2 and child attentional function at 4–5 years of age. *Environment International,* 106**,** 170-177.

Sunyer, J. (2017) Short-term and long-term effects of traffic air pollution on school children neurodevelopment. *Italian Journal of Pediatrics,* 43.

Sunyer, J., M. Esnaola, M. Alvarez-Pedrerol, J. Forns, I. Rivas, M. López-Vicente, E. Suades-González, M. Foraster, R. Garcia-Esteban, X. Basagaña, M. Viana, M. Cirach, T. Moreno, A. Alastuey, N. Sebastian-Galles, M. Nieuwenhuijsen & X. Querol (2015) Association between traffic-related air pollution in schools and cognitive development in primary school children: a prospective cohort study. *PLoS Med,* 12**,** e1001792.

van Kempen, E., P. Fischer, N. Janssen, D. Houthuijs, I. van Kamp, S. Stansfeld & F. Cassee (2012) Neurobehavioral effects of exposure to traffic-related air pollution and transportation noise in primary schoolchildren. *Environ Res,* 115**,** 18-25.

Wang, S., J. Zhang, X. Zeng, Y. Zeng, S. Wang & S. Chen (2009) Association of traffic-related air pollution with children's neurobehavioral functions in Quanzhou, China. *Environ Health Perspect,* 117**,** 1612-8.

Yorifuji, T., S. Kashima, M. H. Diez, Y. Kado, S. Sanada & H. Doi (2017) Prenatal exposure to outdoor air pollution and child behavioral problems at school age in Japan. *Environ Int,* 99**,** 192-198.

Yorifuji, T., S. Kashima, M. Higa Diez, Y. Kado, S. Sanada & H. Doi (2016) Prenatal Exposure to Traffic-related Air Pollution and Child Behavioral Development Milestone Delays in Japan. *Epidemiology,* 27**,** 57-65.
